# Transcriptome analysis of African swine fever virus I9R-mediated modulation of host antiviral immunity

**DOI:** 10.1016/j.virusres.2026.199775

**Published:** 2026-07-06

**Authors:** Yan Lijiao, Zhou Yanlong, Ren Jingjing, Song Daming, Wang Mengyi, Hai Xinqi, Zheng Haixue, Li Dan

**Affiliations:** a College of Veterinary Medicine, Gansu Agricultural University, Lanzhou 730070, China; b State Key Laboratory of Animal Disease Control and Prevention, College of Veterinary Medicine, Lanzhou University, Lanzhou Veterinary Research Institute, Chinese Academy of Agricultural Sciences, Lanzhou 730046, China; c African Swine Fever Regional Laboratory of China (Lanzhou), Gansu Province Research Center for Basic Disciplines of Pathogen Biology, Lanzhou 730046, China

**Keywords:** African swine fever, I9R, Transcriptome sequencing, Immunity

## Abstract

African swine fever (ASF) is an acute hemorrhagic disease caused by African swine fever virus (ASFV), with mortality rates approaching 100% in domestic pigs. Because its genome is large and structurally complex, the functions of many ASFV encoded genes remain poorly understood. In this study, ASFV I9R was identified as a relatively conserved early transcribed gene. To investigate its biological role, an I9R deletion recombinant virus (ASFV-ΔI9R) was generated by replacing the I9R gene with an enhanced green fluorescent protein (EGFP) expression cassette. In primary porcine alveolar macrophages (PAMs), ASFV-ΔI9R and the parental ASFV CN/GS/2018-WT (ASFV-WT) exhibited identical replication kinetics, indicating that I9R is dispensable for viral replication in vitro. Transcriptome sequencing (RNA-seq) analysis of infected PAMs at 18 and 36 hour post infection (hpi) showed that differentially expressed genes (DEGs) in ASFV-ΔI9R-infected cells were mainly enriched in innate immune signaling pathways, particularly pathways associated with type I interferon (IFN-β) mediated antiviral responses. Further analyses demonstrated that ASFV-ΔI9R infection reduced IFN-β-induced the expression of interferon-stimulated gene 12A (ISG12A) and inhibited phosphorylation levels of STAT1 and STAT2, key components of the JAK-STAT signaling pathway. Collectively, these findings suggest that I9R is involved in modulation of host antiviral responses through the JAK-STAT signaling pathway. This study provides additional insights into ASFV host interactions and contributes to understanding the biological functions of ASFV encoded genes.

## Highlight

• ASFV I9R is an early transcription gene.
• ASFV I9R is dispensable for viral replication in vitro.
• ASFV I9R deletion inhibits JAK-STAT signaling and ISG12(A) expression.

## 1 Introduction

African swine fever virus (ASFV) is a member of the Asfarviridae family of nucleocytoplasmic large DNA viruses (NCLDVs) and is the only known DNA insect-borne virus [Akira et al., 2006]. Owing to its exceptional contagiousness, high morbidity, and mortality, ASF is listed as a notifiable disease by the World Organisation for Animal Health (WOAH, founded as the OIE) [Costard et al., 2013]. ASF has spread extensively across many regions worldwide, including East, South, and Southeast Asia, and was introduced into China in 2018, posing a serious threat to global food security and the sustainability of the livestock industry [Gao et al., 2020; Le et al., 2023; Spinard et al., 2023]. The ASFV coding region is between 170 - 193 kbp and encodes 150–200 proteins, including 68 structural proteins and >100 non-structural proteins [Wang et al., 2021]. Interestingly, sequencing of the 5′ ends of viral mRNAs uncovered multiple unannotated open reading frames (ORFs) encoding short polypeptides of 25–56 amino acids (aa), with several of these small ORFs residing within or initiating upstream of previously annotated larger coding sequences [Cackett et al., 2020]. However, the functions of over half of ASFV encoded proteins remain elusive, representing a major bottleneck in rational vaccine design.

The innate immune system constitutes the first line of host defense against invading microbial pathogens and plays a pivotal role in orchestrating antiviral resistance. Germline-encoded pattern recognition receptors (PRRs) detect evolutionarily conserved pathogen-associated molecular patterns (PAMPs), thereby initiating signaling cascades that culminate in the activation of proinflammatory and antimicrobial response [Akira et al., 2006]. Interferon type I (IFN-I) is an essential component of the intrinsic immune response in virtually any nucleated cell, and is considered the major host antiviral pathway [Akira et al., 2006; Dixon, 2025]. IFN-I is secreted by infected cells and acts on itself or other cells to activate the JAK-STAT signaling pathway in bystander or infected cells by signaling through cell surface IFN receptors [Dixon, 2025; MacMicking, 2012]. This process results in the transcriptional activation of numerous IFN-stimulated genes (ISGs) [Gough et al., 2012; Ivashkiv and Donlin, 2013]. These ISGs have multiple roles, including acting as host resistance factors that limit viral replication, activating host innate and adaptive immune responses, and further activating or inhibiting type I IFN responses in feedback loops [Dixon, 2025; Gough et al., 2012; Ivashkiv and Donlin, 2013]. STAT2 was a key component of the JAK-STAT signaling pathway, and interaction with JAK1 leads to phosphorylation of STAT2 (p-STAT2) and formation of a heterodimer with phosphorylated STAT1 (p-STAT1) via the Src homology 2 domain (SH2) [Gough et al., 2012]. Subsequently, its translocation to the nucleus with interferon regulatory factor 9 (IRF9) [Wang et al., 2020; Banninger and Reich, 2004], activating transcription of antiviral cytokines through the interferon-sensitive response element (ISRE) promoter [Platanitis et al., 2019; Philips et al., 2022].

Studies had shown that ASFV can induce the expression of IFN-I and ISGs through the cGAS/STING and RIG-I signaling pathways. ASFV I267L exerts virulence by damaging the innate immune response mediated by the RNA Pol-III-RIG-I axis [Ran et al., 2022]. ASFV MGF-505–7R negatively regulates the cGAS/STING mediated signaling pathway, promotes the expression of autophagy related protein ULK1 to degrade STING, interacts with IRF3 and inhibits its nuclear translocation, thereby blocking the production of IFN-I [Huang et al., 2022; Li et al., 2021]. In addition, ASFV MGF360–4 L could also inhibit the cGAS/STING signaling pathway by suppressing the IFN-β promoter activity induced by cGAS/STING. And it further reduces the transcriptional levels of ISG15, ISG54, ISG56, STAT1, STAT2 and TYK2 [Wang et al., 2024]. Importantly, ASFV could also inhibit the JAK/STAT pathway by interfering with the production of IFN-I. The ASFV-I7L protein exerts its pathogenicity by inhibiting the phosphorylation of STAT1 and antagonizing the JAK-STAT signaling pathway triggered by IFN-γ [Reis et al., 2016]. The pK205R protein interacts with the cytoplasmic domains of IFNAR1 and IFNAR2, preventing the activation of JAK-STAT signaling and thereby evading the host immune system [Huang et al., 2024]. Meanwhile, the ASFV immunosuppressive protein pB475L inhibits IFN-I signaling by suppressing heterodimerization and nuclear translocation of STAT1 and STAT2 [Huang et al., 2024]. In addition, the deletion of some virulence-related genes has been used in the development of ASF attenuated vaccines. The antiviral response mediated by IFN-I plays an important role in resisting pathogen invasion and host immune response [Ivashkiv and Donlin, 2013; Reis et al., 2016]. Therefore, screening ASFV proteins related to the host antiviral response is crucial for disease-resistant breeding and ASF gene deletion vaccines.

In this study, we identified I9R as an early transcribed gene and investigated its potential role during ASFV infection. Further analyses revealed that deletion of I9R inhibited the activation of the JAK-STAT signaling pathway, as evidenced by decreased phosphorylation levels of STAT1 and STAT2, as well as reduced ISG12A transcription following IFN-β stimulation. These findings suggest that I9R can participate in the regulation of type I interferon-stimulated gene expression and host antiviral responses during ASFV infection. This study provides additional insights into the biological function of I9R and ASFV-host interactions.

## 2 Materials and methods

### 2.1 Cells and the virus

PAMs were isolated from three Duroc-landrace-yorkshire swine through bronchoalveolar lavage, as previously described [Carrascosa et al., 1982]. PAMs were cultured in RPMI 1640 (Gibco, manufactured at Thermo Fisher Biochemical Products (Beijing); Beijing, China) and supplemented with 10% fetal bovine serum (Gibco) and 1% penicillin-streptomycin (Thermo Fisher Biochemical Products; Beijing, China) at 37 °C with 5% CO_2_. The current circulating virulent ASFV CN/GS/2018 isolate was characterized and preserved by the Regional Laboratory of ASF, Lanzhou Veterinary Research Institute.

### 2.2 Antibodies and reagents

The rabbit anti-STAT1 monoclonal antibodies (MAb) (14,994), rabbit anti-P-STAT1 (Y701) MAb (9167), rabbit anti-STAT2 MAb (72,604), rabbit anti-p-STAT2 (Y690) MAb (88,410) were purchased from Cell Signaling Technology (Danvers, USA). Rabbit anti-β-actin MAb (AC026) was obtained from ABclonal (Wuhan, China), DyLight 488 goat anti-mouse IgG (A23210), DyLight 405 goat anti-rabbit IgG (A0605) was acquired from Beyotime (Shanghai, China). 4′,6-diamidino-2′-phenylindole (DAPI, C0065) was purchased from Solarbio (Beijing, China). Mouse anti-Flag tag MAb (66,008-4-Ig) was purchased from Proteintech (Chicago, USA). Mouse anti-ASFV-p30 Mab was produced and stored in our laboratory. The mouse IgG (A7028) and rabbit IgG (A7016) were sourced from Beyotime. IFN-β (300–02BC) were procured from PeproTech (Cranbury, NJ, USA).

### 2.3 Plasmids and transfection

The expression plasmids of pRK-I9R-Flag was generated using homologous recombination molecular biology techniques: the I9R gene fragment was amplified from the ASFV genome and cloned into the pRK-Flag vector to generate the eukaryotic expression plasmid.

HEK293T cells were seeded at 2 × 10⁴ cells per well in confocal dishes and cultured until reaching 70% confluence. The plasmid was then transfected into the cells using jetPRIME® transfection reagent (Polyplus-transfection, Illkirch, France; cat. no. 114–15) for subsequent indirect immunofluorescence assay. Control plasmid was added to ensure that each transfection was performed using the same amount of the total DNA.

### 2.4 Construction and identification of an ASFV CN/GS/2018 I9R gene deletion recombination virus

The enhanced green fluorescent protein (EGFP) expression cassette controlled by the ASFV p72 promoter was inserted into the viral genome by using the homologous recombination construction strategy [Zhou et al., 2022] and construct a vector for the ASFV-ΔI9R gene deletion mutant strain. First, the ASFV-WT genome was used as a template to design specific primers containing designated restriction enzyme sites flanking the I9R gene locus. The left and right homologous arms were amplified by PCR. Subsequently, primers were designed using the EGFP reporter gene expression cassette driven by the p72 promoter as the template to amplify the reporter fragment.

The amplified fragments were then mixed with the pUC57 vector at a molar ratio of 1:1:1 and combined with an equal volume of multi-fragment recombinase (Gibson Assembly, E2611; New England Biolabs). The mixture was incubated at 50 °C for 1 hpi to facilitate assembly. The recombinant product was subsequently transformed into *Escherichia coli* DH5α competent cells and plated on ampicillin-containing agar plates. After colony growth, approximately ten single colonies were randomly selected for screening and identification, resulting in the recombinant plasmid pUC57-ASFV CN/GS/2018-ΔI9R. The primers used in this study are listed in Table 1.

**Table 1:** Primers were used to assay for insertion location and purity by PCR.

**Primers**	**Sequence (5–3′)**	**Description**
ΔI9R-LA-F	cttttcctccggcgaccctcactttttgcagaggcagttac	For ASFV-WT
ΔI9R-LA-R	cttttcctccggcgaccctcactttttgcagaggcagttac	
P72-GFP-F	gtttatataaaactaacaaacaAACTTGTTTATTGCAGCTTATAATG	For marker gene
P72-GFP-R	gtaactgcctctgcaaaaagtgagggtcgccggaggaaaag	
ΔI9R-RA-F	CATTATAAGCTGCAATAAACAAGTTtgtttgttagttttatataaac	For ASFV-WT
ΔI9R-RA-R	GACCATGATTACGCCAAGCTCTCGAGctacttggattaaatctattc	
I9R-F	atgggaactttttcagtaac	I9R gene
I9R-R	acatgatgacacaccatgtt	
P-ΔI9R-F	gatatgttactttaaaaacatttg	Detect the purity
P-ΔI9R-R	cataaaggttgcataggatggg	

F, forward; R, reverse.

The recombinant plasmid was then transfected into PAMs before infecting with ASFV-WT. Recombinant viruses were isolated and purified by serial limiting dilution. The purified ASFV-ΔI9R virus was subsequently amplified in PAMs to generate viral stocks. To verify the absence of residual wild-type viral sequences in the recombinant virus, viral DNA was extracted from PAMs infected with the ASFV-ΔI9R strain and subjected to PCR analysis using specific primers (listed in Table 1).

### 2.5 RNA isolation and quantitative real-time reverse transcription PCR(RT-qPCR)

RNA was extracted using TRIzol reagent (ThermoFisher Biochemical Products, Beijing,China). The cDNA was synthesized using a Prime Script RT reagent kit (TaKaRa Bio Inc,Dalian, China) and then used as the template for real-time qPCR using the TB Green PremixEx Taq mix kit (TaKaRa Bio Inc., Dalian, China). RT-qPCR assays were performed on a C1000 Touch Thermal Cycler (Bio-Rad Laboratories, Singapore) and the software used was Bio-Rad CFX Manager 3.1(Bio-Rad Laboratories, Singapore) for 40 cycles of denaturation (96 °C for 5 s), reannealing, and extension (60 °C for 30 s). The relative mRNA level of these genes was normalized to the porcine GAPDH mRNA level. mRNA was calculated and normalized based on the comparative cycle threshold (2^−ΔΔCt^) method. The primers of each gene are shown in Table 2.

**Table 2:** Primers and oligonucleotides used in this study.

**GenBank Number**	**Primers**	**Sequences**
AF141959.1	GAPDH-F	ACATGGCCTCCAAGGAGTAAGA
	GAPDH-R	GATCGAGTTGGGGCTGTGACT
FR682468.2	CP204L-F	CTCCGATGAGGGCTCTTGCT
	CP204L-R	AGACGGAATCCTCAGCATCTT
FR682468.2	B646L-F	TCCGAACTTGTGCCAATCTC
	B646L-R	CAACAATAACCACCACGATGA
XM_021088503.1	GBP1-F	GTGGAACGTGTGAGAGCTGA
	GBP1-R	AGTCGGGCTTGTTCCTGAAG
NM_001198921.1	ISG12A-F	GTAGCCACCCAAGCAGTCTT
	ISG12A-R	CAGCAAGGATCCCAGGCTAG
XM_021073680.1	OAS1-F	CTTTGATGCCCTGGGTCAGT
	OAS1-R	ACGTCTGGTACCAGTGCTTG
NW_026947484.1	DUOX2-F	CGGCTGGAGCTATTCTCTGG
	DUOX2-R	CGAAGGGTGGTGCTTCTGAT
NM_001001624.1	CCR9-F	TGAAGTCGGCTGTCTTGACC
	CCR9-R	TTAGTGGAAACGGCACAGCT
NM_001123091.1	CAV2-F	TTCCTAACGGTGTTCCTGGC
	CAV2-R	ACAGAAGAGAAGCTGCGTCC
XM_005665795.3	LST1-F	TCCTGGTTGTGGTCGTTCTG
	LST1-R	AATGCAGGCGTAGTCCGTAC
XM_003125820.4	HAO2-F	CAGATGTCCCCCATTGACCC
	HAO2-R	AACCTCATCAAGCTGCCTCC

### 2.6 Virus titration

The ASFV-WT and ASFV-ΔI9R viruses were quantified using the hemadsorption (HAD) assay. PAMs were infected with ASFV -WT and ASFV-ΔI9R for the indicated times. The cells and the culture medium were frozen and thawed at −80 °C. This process was repeated three times and then diluted. PAMs were seeded in 96-well plates, and the samples were added to the plates and titrated in triplicate using 10-fold serial dilutions. Fresh suspensions of autologous swine erythrocytes were added to each sample. 50% HAD dose (HAD_50_) was determined on day 7 post-inoculation.

### 2.7 Red blood cell adsorption assay

Whole blood was collected from healthy pigs into anticoagulant-containing tubes, diluted 1:10 with sterile PBS, and mixed thoroughly. An aliquot of the diluted blood was then centrifuged at 300 ×g for 5 min. The supernatant was discarded and the pellet was resuspended in sterile PBS. This centrifugation step was repeated until the supernatant became clear. Then, aliquot the processed whole blood and store it for future use. PAMs were seeded in 48-well culture dishes. Two hours later, 1 MOI of ASFV-WT and ASFV-ΔI9R were infected. Two days after infection, the treated red blood cells were diluted at a ratio of 1:100 and then added to PAMs. After continuing to culture for 3–4 days, the adsorption phenomenon of red blood cells was observed through a fluorescence microscope.

### 2.8 The ASFV-ΔI9R growth curve

PAMs were inoculated in 48-well culture dishes. After 2 hpi, the PAMs were infected with 1 MOI of ASFV-WT and ASFV-ΔI9R. After virus adsorption for 2 hpi, discard the supernatant. Wash the cells three times with PBS and then replace them with fresh culture medium. Cells and culture supernatants were collected at 3, 6, 9, 12, 24, 36, 48 and 72 hpi, and were repeatedly frozen and thawed three times. After centrifugation at 1000 ×*g* in a 4 °C centrifuge for 10 min, the supernatant was collected. The HAD_50_ of the virus were determined, and generate the growth curve using GraphPad Prism. Set three repetitions at each time point.

### 2.9 Viral genome replication test

PAMs were inoculated in 48-well plates. After 2 hpi, the PAMs were infected with 1 MOI of ASFV-WT and ASFV-ΔI9R. The culture medium was discarded after 1 hpi. The cells were washed three times with PBS and fresh culture medium was added and placed in a 37 °C incubator for culture. Cells and culture supernatants were collected at 3, 6, 9, 12, 24, 36, 48 and 72 hpi after infection, respectively. The genomic replication level of ASFV was detected using specific probe primers.

### 2.10 RNA-seq and data analysis

Under the condition of MOI being 1.0, PAMs were infected with ASFV-WT or ASFV-ΔI9R for 18 and 36 hpi respectively. Uninfected PAMs were used as the simulated control group. RNA was extracted from the five treatment groups (non-treated group, ASFV-WT 18 hpi, ASFV-WT 36 hpi, ASFV-ΔI9R-18 hpi and ASFV-ΔI9R 36 hpi). After DNA digestion with DNase, PCR amplification was performed. Build a library. After passing the quality inspection with Agilent 2100 Bioanalyzer, transcriptome sequencing was carried out. The fastp software was used to conduct quality preprocessing on the original data, and the number of reads throughout the quality control process was statistically summarized. The sequencing data statistics of each sample are shown in Table 3. The Q30 base distribution ranges from 97.13% to 97.76%, and the average GC content is 46.73%. By aligning reads onto the reference genome, the genomic alignment of each sample was obtained, with an alignment rate ranging from 61.51% to 97.9%. It can be seen from this that the selected reference genome meets the requirements for subsequent analysis. The RNA-seq analysis data are shown in Table 3.

**Table 3:** The summary of the RNA-Seq raw data.

**Samples**	**RawReads(M)**	**CleanReads(M)**	**ValidBasea(%)**	**Q30(%)**	**GC(%)**
Mock	24.59	23.87	97.05	97.33	50.94
ASFV-WT-18h	24.51	23.88	97.41	97.13	49.93
ASFV-WT-36h	24.53	24.07	97.63	97.68	42.87
ASFV-ΔI9R-18h	24.71	24.00	97.13	97.50	48.21
ASFV-ΔI9R-36h	24.89	24.07	96.69	97.76	41.71

Note: Differentially expressed genes (DEGs) at the peak of lactation and in non-lactating mammary gland parenchyma.

Differentially expressed genes (DEGs) were analyzed using DEGSeq, which has been applied for transcriptomic analysis in datasets. Genes with log2(Foldchange) > 1 and Benjamini-Hochberg-adjusted p value (q value) < 0.05 are regarded as differentially expressed genes [Wang et al., 2009; Klipper-Aurbach et al., 1995]. The ClusterProfiler program was used to conduct GO and KEGG enrichment analyses on the GO database and the KEGG database. And use Fisher's accuracy probability test to calculate the number of differentially expressed genes contained in each GO and KEGG term, and calculate the p-value of each term. The GO and KEGG terms with p values <0.05 were defined as significantly enriched.

### 2.11 Western blotting

The effect of ASFV-ΔI9R infection on the phosphorylation levels of STAT1 and STAT2 was detected by Western blotting. The specific operation is as follows: PAMs are inoculated in 6-well plates and infected with 1 MOI of ASFV-WT and ASFV-ΔI9R 2 hpi later. Twenty-four hours after infection, PAMs were treated with IFN-β (400 ng/mL), while uninfected cells were used as controls. Resuspend the cells with NP40 lysate and add 2×SDS loading, then heat in a 90 °C metal bath for 10–15 min. After vortex mixing, centrifuge at 8000 ×g for 10 min, and take the supernatant for SDS-PAGE electrophoresis. The SDS-PAGE electrophoresis condition was 150 V, and protein transfer was performed after electrophoresis for 1 hpi. Transfer at 100 V for 2 hpi. Following transfer, the membrane was blocked with 5% skim milk either for 30 min at room temperature or overnight at 4 °C. After the blocking was completed, the primary antibody and the corresponding secondary antibody were incubated successively. Subsequently, the proteins were measured using enhanced chemiluminescence (Epizyme, SQ202L-2).

### 2.12 Indirect immunofluorescence assay

HEK-293T cells were inoculated in confocal microdishes and transfected with the empty vector and pRK-I9R-Flag eukaryotic expression vector 12 hpi later. After transfection, the cells were washed three times with PBS 24 hpi later, and then fixed at room temperature with pre-cooled 4% paraformaldehyde for 20–30 min or overnight at 4 °C. After fixation, wash the cells three times with PBS, add 0.2%−0.3% triton to allow them to permeate at room temperature for 10 min, and then wash them three times with PBS. Cells were blocked with 5% BSA at room temperature for 1 hpi, and then washed three times with PBS. Anti-flag mouse monoclonal antibody (diluted 1:500) was added and incubated overnight at 4 °C. After incubation, cells were washed three times with PBST. Subsequently, Alex labeled murine fluorescent secondary antibody (diluted 1:500) was added and incubated at room temperature in the dark for 1 hpi. Wash the cells three times with PBST. Finally, DAPI staining solution was added to stain the cell nuclei and incubated at room temperature for 10 min. After staining, the cells were washed with PBST, and then the cells were observed and images were collected using a Leica TCS SP5 II AOBS confocal microscope.

### 2.13 Facility biosafety statement

All ASF live virus experiments were carried out in the biosafety level 3 (P3) facility of Lanzhou Veterinary Research Institute of the Chinese Academy of Agricultural Sciences and approved by the Ministry of Agriculture and Rural Affairs and the China National Accreditation Service for Conformity Assessment.

### 2.14 Statistical analysis

All graphs were generated using GraphPad Prism 8.0 (USA). Statistical comparisons between groups were performed with one-way analysis of variance (ANOVA). Differences were considered statistically significant when * p < 0.05, ** p < 0.01, or *** p < 0.001, and non-significant when p > 0.05.

## 3 Results

### 3.1 ASFV I9R is an early transcription gene

**Fig. 1:**
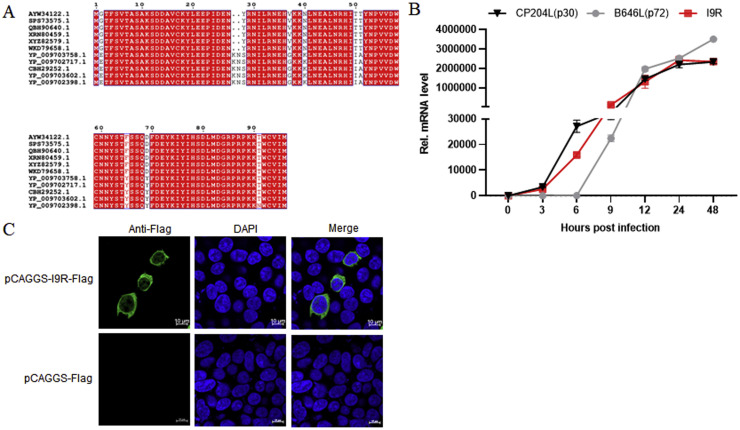
ASFV I9R is an early transcription gene. (A) Amino acid sequence alignment of ASFV I9R. The amino acid sequences of I9R from 10 African swine fever virus strains were compared to characterize the pattern of multi-residue substitutions by MEGA5.1 software. (B) The expression levels of I9R, CP204L, and B646L mRNA. RT-qPCR was performed on I9R, CP204L, B646L and GAPDH using specific primers to detect the average cycle thresholds of ASFV-WT infected primary porcine alveolar macrophages (MOI of 1.0) at 0, 3, 6, 9,12,24 and 48 hpi. (C) Localization of pI9R protein in HEK293T cells. Under confocal microscopy, HEK293T cells were transfected with pCGAAS-I9R-Flag. At 24 hpi, the cells were incubated with mouse anti-flag and Alexa Fluor 488(green) conjugated secondary antibodies, respectively. The nuclei (blue) were stained with 4′, 6-diamino-2-phenylindole (DAPI). bar, 10 μm.

Previous studies had shown that deletion of the I9R gene does not affect viral replication or virulence; however, the function of this gene remains uncharacterized [Fan et al., 2024]. To explore the function of the I9R gene, amino acid sequence homology analysis of I9R showed that the sequence similarity of I9R was 98% in indicated isolated strains by the MEGA 5.1 software (Fig. 1A), suggesting that I9R was highly conserved in these isolates. Meanwhile, reverse transcription quantitative PCR (RT-qPCR) was performed to evaluate the transcriptional kinetics of I9R relative to established temporal markers: the early gene CP204L (encoding p30) and the late gene B646L (encoding p72). The results demonstrated that I9R transcript levels increased rapidly between 3 and 6 hpi, exhibiting an expression pattern comparable to that of the early gene CP204L (Fig. 1B), suggesting that I9R is an early transcribed gene during ASFV infection. Furthermore, indirect immunofluorescence assay of cells transfected with pCAGGS-I9R-Flag confirmed that I9R localizes to the cytoplasm (Fig. 1C).

### 3.2 Construction of the deletion of ASFV I9R gene recombinant virus

**Fig. 2:**
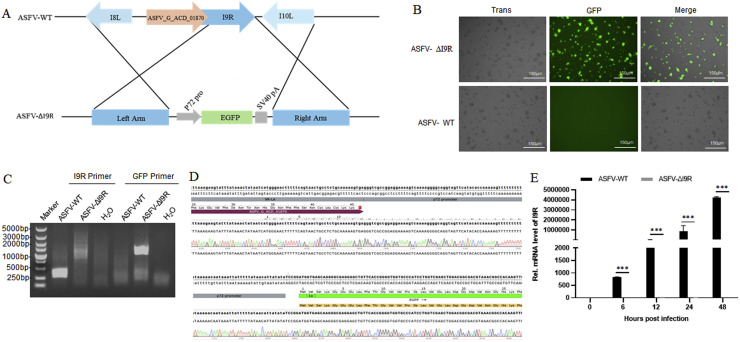
Construction of ASFV-∆I9R. (A) Schematic diagram of the construction of I9R deletion mutant. (B) Infection of PAMs with purified ASFV-∆I9R, scale, 150 µm. (C) PCR amplification confirmed the deletion of the ASFV I9R gene. (D) Sequencing results for the region covering the homologous recombination site of the ASFV-ΔI9R strain. (E) Cells were infected with ASFV-WT or ASFV-∆I9R (MOI = 1) respectively, and samples were collected at 6, 12, 24 and 48 hpi after infection respectively. RNA was extracted by quantitative reverse transcription PCR (qRT-PCR) and the transcriptional level of the I9R gene was detected using I9R primers.

To determine the role of the I9R gene during ASFV infection, a recombinant ASFV strain lacking the I9R gene (ASFV-ΔI9R) was constructed (Fig. 2A). The recombinant virus replication was validated by monitoring EGFP fluorescence signals in infected PAMs (Fig. 2B). After seven rounds of limiting dilution purification of ASFV-ΔI9R, viral purity was verified by PCR amplification using I9R- and EGFP-specific primers. The results showed that the recombinant virus ASFV-ΔI9R contained the p72-EGFP cassette but lacked the I9R gene sequence, indicating the successful generation of the ASFV-ΔI9R strain (Fig. 2C). To confirm that the I9R gene had been accurately deleted, we sequenced the region covering the homologous recombination site. The sequencing results confirmed that the I9R open reading frame had been precisely replaced by the EGFP expression cassette, and no additional mutations were detected within the analyzed region, further confirming the successful construction of the ASFV-ΔI9R strain (Fig. 2D).

To characterize the transcription profile of the I9R gene, total RNA was extracted from ASFV-WT or ASFV-ΔI9R-infected PAMs at indicated times, and I9R transcription was analyzed. I9R transcripts were first detected in ASFV-WT-infected cells at 6 hpi, and the transcription level progressively increased at later time points. In contrast, no I9R transcripts were detected in ASFV-ΔI9R-infected cells throughout the infection period (Fig. 2E). These results confirmed the successful deletion of the I9R gene.

### 3.3 Deletion of I9R does not affect ASFV replication in PAMs

**Fig. 3:**
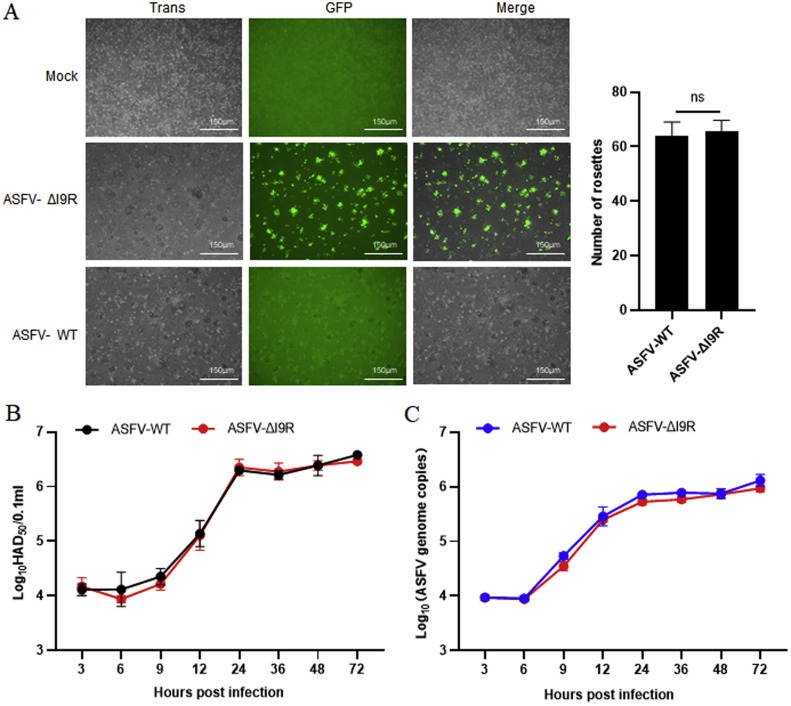
Deletion of I9R does not affect ASFV replication in PAMs. (A) HAD_50_ assay. PAMs were infected with ASFV-ΔI9R or the parental ASFV-WT strain. At 24 hpi, whole blood from healthy pigs was added. Hemadsorption rosettes formed by red blood cells were observed in PAMs infected with ASFV-WT or ASFV-ΔI9R. In addition, green fluorescence was detected in ASFV-ΔI9R-infected cells, indicating the expression of EGFP. No hemadsorption or fluorescence signal was observed in uninfected control cells. (B) Growth kinetics analysis. PAMs were infected with ASFV-WT or ASFV-ΔI9R at MOI of 1. Samples were collected at 3, 6, 9, 12, 24, 36, 48, and 72 hpi. Viral titers were determined using the HAD₅₀ assay. (C) Viral genome replication analysis. PAMs were infected with ASFV-WT or ASFV-ΔI9R (MOI = 1), and samples were collected at 3, 6, 9, 12, 24, 36, 48, and 72 hpi. Viral genome replication levels were determined by quantitative PCR.

To determine whether deletion of the I9R gene affects the biological characteristics of ASFV, a hemadsorption (HAD) assay was conducted. Characteristic hemadsorption “rosette” formations, in which erythrocytes adhered to the surface of PAMs, were observed in both ASFV-ΔI9R- and ASFV-WT-infected cells, indicating that deletion of the I9R gene did not affect the hemadsorption phenotype of ASFV. No significant difference in the number of rosettes was detected between the two groups, indicating that deletion of the I9R gene does not affect the hemadsorption ability of ASFV (Fig. 3A). To assess whether differences in replication levels exist between the recombinant strain ASFV-ΔI9R and the parental strain ASFV-WT in PAMs, multistep growth curve were generated for both strains using HAD₅₀ assay. The growth profile of ASFV-ΔI9R was similar to that of ASFV-WT in PAMs within 72 hpi indicating that the deletion of the I9R gene does not affect the replication of ASFV in PAMs. (Fig. 3B). Consistently, analysis of viral genome replication showed no significant difference between ASFV-ΔI9R and ASFV-WT. These results indicate that deletion of the I9R gene does not affect the replication capacity of ASFV in PAMs, suggesting that I9R is dispensable for ASFV replication in vitro (Fig. 3C).

### 3.4 Gene ontology analysis of DEGs

**Fig. 4:**
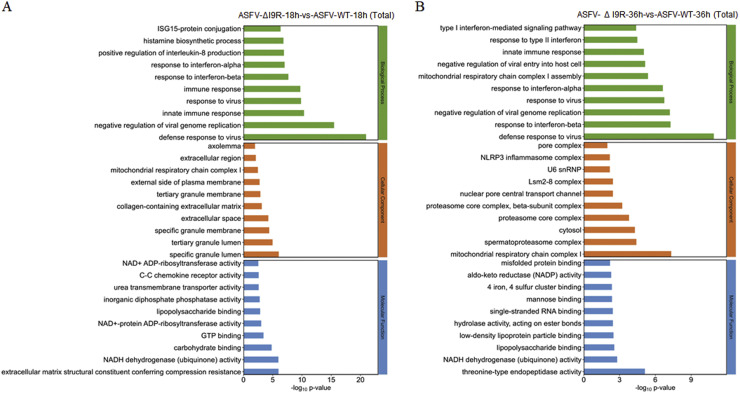

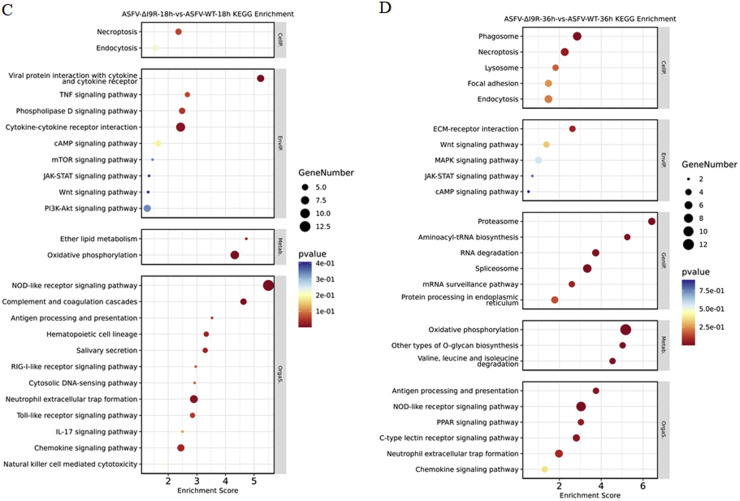
GO and KEGG analysis of ASFV-∆I9R-infected PAMs through RNA-seq. (A) GO analysis of ASFV-∆I9R vs. ASFV-WT at 18 hpi. (B) GO analysis of ASFV-∆I9R vs. ASFV-WT at 36 hpi. (C) KEGG analysis of ASFV-∆I9R vs. ASFV-WT at 18 hpi. (D) KEGG analysis of ASFV-∆I9R vs. ASFV-WT at 36 hpi.

To further investigate the biological function of the I9R gene, RNA-seq analysis was performed in this study. Gene Ontology (GO) functional enrichment analysis comprises three major categories: biological processes (BP), cellular components (CC), and molecular functions (MF), allowing gene functions to be characterized from multiple perspectives. The results showed that DEGs in the BP category were mainly involved in host antiviral immune activation and defense responses to viral infection at both 18 and 36 hpi. These processes included the IFN-I-mediated signaling pathway, response to type II interferon, innate immune response, immune response to virus, response to interferon-beta, negative regulation of viral entry into host cells, negative regulation of viral genome replication, and defense response to virus. Importantly, DEGs were enriched in components such as the extracellular region of the plasma membrane, the NLRP3 inflammasome complex, the proteasome core complex, and mitochondrial respiratory chain complex I in the CC and MF categories. These enriched components collectively constitute the molecular basis of host antiviral defense (Figs. 4A and 4B).

### 3.5 Kyoto encyclopedia of genes and genomes (KEGG) pathway enrichment analyses

To further investigate the impact of I9R on host biological processes, KEGG pathway enrichment analysis was performed. At 18 hpi, DEGs were significantly enriched in several immune-related pathways, including the RIG-I-like receptor signaling pathway, Toll-like receptor signaling pathway, NOD-like receptor signaling pathway, JAK-STAT signaling pathway, TNF signaling pathway, natural killer cell-mediated cytotoxicity, and protein processing in the endoplasmic reticulum (Fig. 4C). At 36 hpi, DEGs were mainly enriched in the chemokine signaling pathway, JAK-STAT signaling pathway, ECM-receptor interaction, proteasome, NOD-like receptor signaling pathway, and neutrophil extracellular trap formation (Fig. 4D). Notably, pathways such as the JAK-STAT signaling pathway, NOD-like receptor signaling pathway, neutrophil extracellular trap formation, and chemokine signaling pathway were enriched at both time points. Those data indicated that I9R plays a crucial role in inducing innate immunity.

### 3.7 Transcriptome validation of host genes

**Fig. 5:**
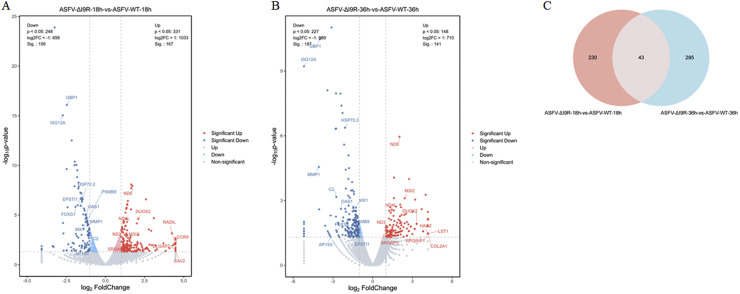
Analysis of differentially expressed genes. (A) Volcano plot showing DEGs between ASFV-ΔI9R infected and ASFV-WT infected PAMs at 18 hpi. (B) Volcano plot showing DEGs between ASFV-ΔI9R infected and ASFV-WT infected PAMs at 36 hpi. (C) Venn diagram showing DEGs between ASFV-ΔI9R infected and ASFV-WT infected PAMs at 18 and 36 hpi. The pink area represents DEGs identified in ASFV-ΔI9R infected vs. ASFV-WT infected PAMs at both 18 and 36 hpi; the red area represents DEGs identified only at 18 hpi; and the blue area represents DEGs identified only at 36 hpi.

**Fig. 6:**
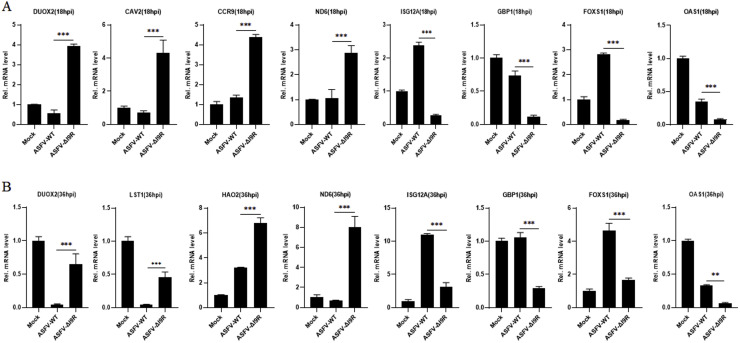
Validation of RNA-seq results by RT-qPCR. (A and B) PAMs were either uninfected or infected with ASFV-WT or ASFV-ΔI9R at MOI of 1. Cells were collected at 18 and 36 hpi, and total RNA was extracted for RT-qPCR analysis to determine gene expression levels. Data are presented as mean ± SD. Statistical significance was analyzed using one-way analysis of variance (ANOVA) Student’s *t*-test. **p < 0.01, ***p < 0.001, ****p < 0.0001; ns, not significant (p > 0.05).

To explore the role of I9R in host innate immune responses, DEGs were analyzed and visualized using volcano plots between ASFV-WT and ASFV-ΔI9R infected PAMs. Compared with the ASFV-WT infection group, a total of 273 DEGs were identified at 18 hpi, including 167 upregulated genes and 106 downregulated genes (Fig. 5A). At 36 hpi, 328 genes were differentially expressed, of which 141 were upregulated and 187 were downregulated (log2FC ≥ 1 or ≤ −1, P < 0.05) (Fig. 5B). In addition, 43 genes were found to be differentially expressed at both 18 h and 36 h post infection (Fig. 5C). Among these, the most significantly downregulated genes included ISG12A, GBP1, OAS1, and FOXS1, whereas ND6 and DUOX2 were among the genes that showed significant upregulation. To validate the RNA-seq results, RT-qPCR analysis was performed using RNA extracted from PAMs infected with ASFV-WT or ASFV-ΔI9R. The results showed that, at both 18 and 36 hpi, the transcription levels of ISG12A, GBP1, OAS1, and FOXS1 were significantly decreased in the ASFV-ΔI9R infection group compared with the ASFV-WT group. In contrast, the transcription levels of ND6 and DUOX2 were significantly increased. In addition, the transcription levels of CCR9 and CAV2 were elevated at 18 hpi, whereas LST1 and HAO2 were upregulated at 36 hpi. These results were consistent with the RNA-seq data (Figs. 6Aand 6A).

### 3.8 Deletion of the ASFV I9R gene suppresses IFN-β-induced innate immune responses

**Fig. 7:**
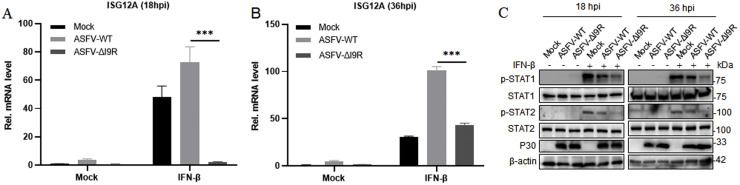
Deletion of I9R suppresses host innate immune responses during ASFV infection. (A and B) PAMs were infected with ASFV-WT or ASFV-ΔI9R at 1 MOI. At 18 and 36 hpi, cells were treated with 400 ng/mL IFN-β for 6 hpi. Total RNA was extracted using TRIzol reagent, and the transcription level of ISG12A was determined by RT-qPCR. Error bars represent the standard error of the mean. (C) PAMs were infected with ASFV-WT or ASFV-ΔI9R (MOI = 1). At 18 and 36 hpi, cells were treated with 400 ng/mL IFN-β for 6 hpi, followed by Western blot analysis using the indicated antibodies. Statistical significance was analyzed using one-way analysis of variance (ANOVA). **p < 0.01, ***p < 0.001, ****p < 0.0001; ns, not significant (p > 0.05).

The IFN-I signaling pathway is a key antiviral defense mechanism in which IFN-I produced by infected cells binds to cell-surface receptors, activates the JAK-STAT pathway, and induces the transcription of numerous interferon-stimulated genes (ISGs) [Dixon, 2025]. To evaluate the effect of I9R on IFN-β-mediated signaling, PAMs were infected with ASFV-WT or ASFV-ΔI9R. The results showed that IFN-β treatment significantly upregulated the transcriptional level of ISG12A in uninfected PAMs, indicating that the expression of ISG12A was responsive to IFN-β. However, compared with ASFV-WT infection, ASFV-ΔI9R infection markedly reduced IFN-β induced ISG12A transcription (Figs. 7A and 7B), suggesting that I9R promotes IFN-β induced ISG12A expression. Previous studies have demonstrated that phosphorylation of STAT1 and STAT2 is essential for activation of the JAK-STAT signaling pathway [Platanias, 2005; Lukhele et al., 2019]. To determine whether I9R influences IFN-β-induced STAT phosphorylation, Western blot analysis was performed. The results showed that, compared with ASFV-WT infection, ASFV-ΔI9R infection significantly reduced IFN-β induced phosphorylation of STAT1 and STAT2 in PAMs at both 18 and 36 hpi (Fig. 7C). Together, these results indicate that deletion of the I9R gene significantly suppresses IFN-β induced activation of the JAK-STAT signaling pathway, suggesting that I9R contributes to the IFN mediated antiviral response during ASFV infection.

## 4 Discussion

In this study, we identified the ASFV I9R gene as an early transcribed gene that is dispensable for viral replication in PAMs. ASFV-ΔI9R exhibited replication kinetics comparable to those of the parental ASFV-WT strain, which is consistent with previous reports showing that simultaneous deletion of the I7L-I11L gene cluster does not impair ASFV replication in vitro [Fan et al., 2024; Zhang et al., 2021]. Although I9R is not essential for viral replication in PAMs, its conservation among different ASFV strains suggests that it may contribute to virus–host interactions during infection. Interestingly, the SY18ΔI7L-I11L mutant reduces viral virulence, protects pigs against virulent ASFV-SY18 challenge, and induces increased IFN-γ production in pigs [Zhang et al., 2021], suggesting that one or more proteins within this gene cluster may participate in regulation of host antiviral responses. Previous studies have shown that pI10L inhibits the NF-κB signaling pathway by targeting IKKβ [Chen et al., 2023], whereas pI7L negatively regulates IFN-γ triggered JAK-STAT signaling by inhibiting STAT1 phosphorylation and homodimerization [Li et al., 2024]. However, the functions of I8L, I9R, and I11L remain largely unclear. Therefore, this study focused on the functional characterization of I9R using RNA-seq and related validation experiments.

Transcriptome analysis demonstrated that deletion of I9R altered host transcriptional responses, with differentially expressed genes predominantly enriched in innate immune signaling pathways, particularly those associated with IFN-I mediated antiviral responses. KEGG pathway analysis further revealed temporal differences in host responses following ASFV-ΔI9R infection. At 18 hpi, DEGs were mainly enriched in pattern recognition receptor signaling pathways, including NOD-like receptor, RIG-I like receptor, and Toll-like receptor signaling pathways, which are associated with early innate immune responses to viral infection. At 36 hpi, DEGs were more closely associated with cellular remodeling and metabolic processes, suggesting dynamic changes in host responses during infection.

Further analyses showed that ASFV-ΔI9R infection reduced phosphorylation levels of STAT1 and STAT2 following IFN-β stimulation and decreased ISG12A transcription. Since STAT1 and STAT2 are key transcription factors in the JAK-STAT signaling pathway, these findings suggest that I9R may participate in modulation of JAK-STAT mediated antiviral signaling. Previous studies have demonstrated that several ASFV proteins target components of the IFN signaling pathway, including pI215L, pDP96R, pA151R, pE301R, and pMGF505–7R [Dodantenna et al., 2022; Ranathunga et al., 2023; Chen et al., 2025, Dodantenna et al., 2024; Riera et al., 2023; Huang et al., 2023]. In addition, ASFV p22 inhibits JAK-STAT signaling by degrading IFN-I receptors [Ren et al., 2025], while pK205R interferes with IFNAR1 and IFNAR2 activation [Huang et al., 2024]. However, the direct molecular target of I9R and the precise mechanism underlying its effects on STAT1/2 phosphorylation remain unclear and require further investigation.

With the recent emergence of genotype I and genotype I/II recombinant strains in Asia and Europe, the development of effective commercial vaccines has become increasingly urgent. However, the large and complex double-stranded DNA genome of ASFV presents a major challenge for vaccine development [Dixon, 2025; Alonso et al., 2018]. Numerous ASFV encoded proteins perform multiple roles in viral replication, transmission, and immune evasion, yet the pathogenic mechanisms of many of these proteins and their interactions with the host immune system remain poorly understood. Consequently, the targeted deletion of immune-evasion-related genes, together with the identification of host antiviral factors, has become a fundamental strategy for the rational design of live attenuated vaccines against ASF.

In conclusion, this study provides the first functional characterization of ASFV I9R and demonstrates that I9R is a non-essential early transcribed gene associated with regulation of host antiviral responses through the JAK-STAT signaling pathway. These findings expand current understanding of ASFV-host interactions and provide a basis for future studies investigating the biological functions of ASFV encoded genes.

## CRediT authorship contribution statement

**Yan Lijiao:** Writing – original draft, Validation, Methodology, Investigation. **Zhou Yanlong:** Methodology, Investigation, Formal analysis. **Ren Jingjing:** Investigation, Formal analysis. **Song Daming:** Methodology, Formal analysis. **Wang Mengyi:** Methodology, Formal analysis. **Hai Xinqi:** Methodology, Formal analysis. **Zheng Haixue:** Supervision, Project administration. **Li Dan:** Writing – review & editing, Writing – original draft, Supervision, Conceptualization.

## Declaration of competing interest

The authors declare that they have no known competing financial interests or personal relationships that could have appeared to influence the work reported in this paper.

## Data Availability

Data will be made available on request.
